# Fathers’ perceived role, self-efficacy and support needs in promoting positive nutrition and physical activity in the first 2000 days of life: a mixed methods study

**DOI:** 10.1186/s12966-024-01575-w

**Published:** 2024-02-26

**Authors:** Mathew Gaynor, Karen Wynter, Kylie D. Hesketh, Penelope Love, Rachel Laws

**Affiliations:** 1 Institute for Physical Activity and Nutrition, School of Exercise and Nutrition Sciences, Deakin University, Geelong, VIC Australia; 2 Centre for Women’s and Children’s Mental Health, Department of Psychiatry, Monash University, Clayton, VIC Australia; 3 School of Nursing and Midwifery, Deakin University, Geelong, VIC Australia

**Keywords:** Fathers, Diet, Physical activity, Early childhood

## Abstract

**Background:**

The first 2000 days of life are a crucial and opportunistic time to promote positive dietary and physical activity behaviours that can continue throughout life. The bulk of research on the impact of parents promoting positive dietary and physical activity behaviours has been on mothers, with the impact of fathers rarely investigated. The aim of this study is to investigate fathers’ perceived role, self-efficacy and support needs in promoting positive dietary and physical activity behaviours in early childhood.

**Methods:**

A sequential explanatory mixed methods study design consisted of a cross sectional survey of Australian fathers (*n* = 200) from a convenience sample, followed by semi-structured qualitative interviews (*n* = 21) with a purposeful sample of Australian fathers.

**Results:**

Quantitative survey data revealed that more than 90.0% of fathers agreed that it is important to role model healthy eating and participating in physical activity with their babies, toddlers and children. A majority of fathers were confident in getting their child to eat fruit/ vegetables (90%) and playing with their child (80%). When searching for information about nutrition and physical activity, the highest proportion of fathers nominated online sources (52%) as their preferred source in survey data. Qualitative interview data revealed that while fathers exhibited high self-efficacy in their abilities, this was susceptible to deterioration due to feelings of isolation, pressures of fatherhood, a lack of information and resources that are father specific, and difficulties navigating the different types of information/resources to find what is right for them.

**Conclusions:**

Although possessing self-efficacy, being committed and seeking knowledge, many fathers found that useful information was hard to find and understand. Appropriate resources are therefore required to support the specific needs of fathers to promote positive dietary and physical activity behaviours in their infants and young children.

**Supplementary Information:**

The online version contains supplementary material available at 10.1186/s12966-024-01575-w.

## Introduction

The first two thousand days of life, from conception to age five, is a period when substantive learning about health-related behaviours occurs that continue throughout life [1]. As health-related behaviours become more difficult to change with age [2], this period is increasingly being recognised as a crucial and opportunistic time to promote positive behaviours, such as healthy diets and physical activity [3, 4]. It is therefore vital that interventions start as early as possible to prevent negative lifestyle patterns becoming entrenched and leading to the development of chronic diseases such as obesity later in life [5, 6]. Internationally however, children are showing lifestyle patterns of decreasing physical activity in outdoor environments, increasing trends of physically restrictive play indoors characterised by device use, low fruit and vegetable intake and consumption of non-core foods that are conducive to the formation of obesity [7–9].

Parents have a crucial role in shaping children’s long-term health behaviours by what they provide, promote and role model [2, 10]. To date, the majority of the focus on parental influences on early childhood diet and physical activity has been on mothers [11]. However, emerging domestic and international trends of increased rates of maternal participation in the workforce, has necessitated more shared responsibilities in child rearing in the home environment [12, 13]. Indeed, recent research of Australian households reveals that over 90% of fathers take responsibility for some of their young children’s meals [14].

Of the limited literature available on fathers, a growing body of evidence indicates that fathers can have a positive influence on dietary and physical activity behaviours in early childhood [15–17]. In a recent systematic review of 23 studies, the eating habits of fathers were strongly associated with their children’s food intake [17]. Similarly, a cross sectional study of 150 fathers of pre-school aged children revealed positive associations between the vigorous physical activity of the fathers and that of their children [16]. Albert Bandura’s social cognitive theory (SCT) suggests that parental behaviours influence children’s health related behaviours as a result of interactions between parental beliefs [18, 19], self-efficacy factors [18, 20] and the social and physical environment [19, 21]. Evidence on fathers’ beliefs about their role in dietary and physical activity behaviours in early childhood is limited to general paternal feeding practices, with positive associations being shown between perceived paternal responsibility for feeding and quantity of meals eaten with family [19, 22]. To our knowledge, fathers’ beliefs about their role in physical activity (and active play) in early childhood has not been studied. Similarly, paternal self-efficacy research is limited to more general parenting roles rather than impacts on early childhood dietary and physical activity behaviours [4, 23, 24].

Despite a growing awareness of the positive effect of fathers on child health-related behaviours, multiple barriers exist preventing engagement of fathers [25–27]. These include a lack of father-focused material and services [28–30], lack of trained staff to work with fathers [14] and limited father-specific, best practice guidelines/advice [31]. Qualitative research in Australia highlights that while fathers are motivated to engage with and promote positive health behaviours to their young children, there is a lack of information that is specific and practical for them [32, 33]. Additionally, Swedish research indicates that fathers can feel misunderstood by health professionals when seeking guidance [34]. Providing parents with high quality information and support to inform healthy family lifestyle behaviours is critical during early childhood, where concern about the child’s growth and development is a constant source of parental stress [35]. Therefore, the aim of this study is to gain an understanding of fathers’ perceived roles, self-efficacy and support needs in promoting positive dietary and physical activity behaviours in the first 2000 days of life.

## Methods

### Study design

A sequential explanatory mixed methods design was used, whereby quantitative data in the form of a cross sectional survey with fathers from a convenience sample preceded qualitative interviews with a purposeful sample. The overall methodology was informed by the flexibility of a pragmatic research paradigm, which provides the underlying philosophical framework for mixed methods research [36]. In allowing for different methods and worldviews to be represented [37], the authors recognise that parts of the resultant data will be socially constructed between the researchers and participants.

### Recruitment

#### Survey

The survey was open to fathers or expectant fathers (18 years or older) with children aged five years or less, residing in Australia and with fluency in English. Participants were recruited using paid Facebook advertising between July and August 2022. Advertisements targeted men aged 18–50 years residing in Australia with an interest in parenting, being an expectant or new parent (of baby aged 0–12 months), parents with toddlers (aged 1–2 years) or parents with pre-schoolers (aged 3–5 years). The survey was distributed by secure Qualtrics software (Provo, Utah, 2020) and was closed when the target sample of 200 survey participants was achieved [38]. A $50 shopping (supermarket/ hardware) vouchers were awarded to five survey participants by random draw.

Within two weeks of completing the survey, a subset of participants was invited to re-take a condensed version of the survey for a test-retest reliability assessment for items that did not have pre-existing, established reliability. Responses from both time points were analysed for correlation which was considered sufficient if *r* ≥ 0.70 [39].

#### Interviews

At the completion of the survey, participants were asked if they would be interested in participating in an interview. Of those expressing an interest, a purposeful sample was invited by email to participate. In line with the complex causes of chronic health conditions such as obesity in children [9, 40–42], a heterogenous sample of fathers was sought, including differing education levels, sociodemographic characteristics, postcode demographics (urban, regional or rural) and number of previous children. To explore the differing role perceptions, self-efficacy and support needs of fathers across the early childhood period, participants included expectant fathers, and fathers of infants, toddlers and preschool children.

The final number of interview participants was guided by the consistency of the qualitative data [43], and was ceased when no new information was elicited, indicating that data saturation had been reached [44]. All interview participants were provided with a $25 shopping voucher.

### Data collection

#### Survey

Potential participants completed online eligibility screening on Qualtrics, and if eligible provided consent before proceeding to the main survey. A combination of existing and modified survey questions was used to explore three domains - perceived parental role, paternal self-efficacy and forms of support.

#### Perceived paternal role

Twelve questions were adapted from the validated Hoover-Dempsey and Sandler instrument [45] which assesses parental (mothers’ and fathers’) role construction for involvement in the child’s day-to-day education. Hoover-Dempsey and Sandler’s models provides a framework to examine specific predictors of parental involvement [23]. Nutrition and physical activity questions were adapted from the original achievement related questions. Questions included such items as “dads should be as involved as the mother in establishing and reinforcing mealtime rules” (see supplementary file 1 for full question list). For scoring, items were collapsed into four categories: (1) strongly/ somewhat disagree, (2) neutral, (3) somewhat agree (4) strongly agree. In line with the original instrument, scores for the three subscales were summed and divided by the number of items to give an average subscale score.

#### Paternal self-efficacy

Twenty questions from a Parental Self-efficacy instrument developed by the Melbourne Infant Feeding Activity and Nutrition Trial (henceforth referred to as the InFANT Program) asked fathers about their self-efficacy in promoting positive dietary and physical activity behaviours [46]. Questions asked fathers about their efficacy in such areas as “get my child to eat a wide range of foods” (see supplementary file 2 for full question list). For scoring, items were collapsed into four categories: (1) not/slightly confident, (2) neutral, (3) somewhat confident, (4) extremely confident. In line with the original instrument, scores for the four subscales were summed and divided by the number of items to give an average subscale score.

#### Forms of support

As no scale exists to measure the use of supports by fathers promoting positive dietary and physical activity behaviours in early childhood, items were adapted from the standardised Physical Activity and Social Support Scale (PASSS) instrument. This scale examines the types of support that affect adult males and females (aged 18–75) in shaping their behaviour towards physical activity [47]. Items were adapted to examine social support that fathers may seek in promoting positive health behaviours in early childhood. Nutrition questions were adapted from the original physical activity questions. Questions included “I talk to friends/family for advice to improve play / physical activity time with child”. Nominated support items included: family, female/ male friends, websites, smart phone apps, social media, education programs, health professionals, childcare staff and other. Ratings of these items were retained as: (1) not beneficial (2) somewhat (3) moderately (4) somewhat beneficial (5) very beneficial. A support score was obtained for each support type which represented the proportion of fathers who used each support type.

Subscales for each of the three scales showed acceptable reliability compared to the original instruments; Cronbach’s α ranged from 0.65 to 0.78. Test re-test reliability was adequate for each of the three scales, ranging from 0.70 to 0.79 [39] (Table 1).

**Table 1:** Survey Domains and Scales

Domain	Scale	Subscales	Items	Response Options	Cronbach’s alpha -Validation^1^	Cronbach’s alpha -Current^2^	Test- retest reliability
Role beliefs	Hoover-Dempsey and Sandler scale	1.Role activity beliefs - Physical activity	4	5-point Likert scale – strongly disagree [1] to strongly agree [5].	0.80	0.78	0.71
		2.Role activity beliefs - Nutrition	4		0.80	0.78	
		3. Partnership focused beliefs	4		0.85	0.66	
Self- efficacy	INFANT	1.Promote healthy eating	8	5 - point Likert scale - not at all confident [1] to extremely confident [5]	0.81	0.73	0.79
		2.Limit unhealthy eating	3		0.86	0.72	
		3.Promoting positive physical activity	6		0.84	0.78	
		4.Limit sedentary behaviour / device use	3		0.86	0.65	
Supports	Physical Activity and Social Support Scale (PASSS)	1.Supports – Physical Activity	11	5 - point Likert scale - not at all beneficial [1] to very beneficial [5].	0.90	0.74	0.70
		2.Supports – Nutrition	11			0.68	

^1^ Subscale internal reliability of original scale; ^2^ Subscale internal reliability in current study

### Sample characteristics

Sociodemographic details collected included fathers’ and children’s date of birth, country of birth, Indigenous status, residential postcode, highest level of education, main language spoken at home, marital and employment status. Father’s body mass index (BMI) was calculated as (kg)/height (m^2^), using self-reported weight and height.

### Interviews

A broad, semi-structured interview used open questions to capture fathers’ role perceptions, self-efficacy and support needs in promoting healthy behaviours in early childhood (see supplementary file 3). Interviews were conducted between August and October, 2022 and were recorded with consent, transcribed via an online platform (zoom) and then manually checked for accuracy by the interviewer. A total of 3 reminder emails were sent at weekly intervals to remind participants to contact the research team.

### Data analysis

#### Survey

IBM SPSS Statistics 28 software (Armonk, NY, 2022) was used to descriptively analyse the survey data. Demographic statistics were calculated as mean (standard deviation, range), percentage or number as appropriate.

#### Interview

Braun and Clarke’s [48] 6-step method of reflexive thematic analysis was used to identify patterns and themes within the interview data. To ensure that the distinct perspective of fathers was captured during coding, a combination of deductive and inductive coding was used [43]. Initially, a top-down, theoretical analysis was completed with the constructs of social cognitive theory as a guide [24]. The data was then coded inductively as the assumptions and ideologies of the data presented [49]. All transcripts were coded by the first author, and the coding framework was discussed with other researchers with a sample of two transcripts. The interviews were ceased when data saturation was established [44]. NVivo 11 software (QSR International Pty Ltd, 2015) was used for coding and retrieval of data [50].

### Researcher reflexivity and credibility

MG is a PhD candidate and a registered psychologist. MG is also a father of a young child and is motivated in role modelling positive health behaviours. MG was conscious that some fathers will not share his knowledge and attitude towards the development of healthy behaviours and was careful not to judge participants in interviews. Other than for administrative purposes, no relationship was established between the researcher(s) and participants prior to the study commencing.

### Ethical approval

This study was approved by the Deakin University Human Research Ethics Committee (HEAG-H 30_2022).

## Results

### Characteristics of participants

A total of 288 people accessed the survey. Of these, five respondents were not eligible, 11 opted to not continue after reading the plain language statement (PLS) and a further 72 did not complete the survey and were excluded from analysis. Respondents took an average of 32 min to complete the 161 questions of the survey. After the target sample of 200 completed surveys was achieved, recruitment was ceased.

Ninety-seven survey respondents indicated interest in the interview. Twenty-eight fathers were purposefully sampled and emailed interview invitations. Of these, 21 fathers responded and completed the interview, which took an average of 44 min to complete. Characteristics of survey and interview participants are presented in Table 2.

**Table 2:** Characteristics of fathers participating in the survey and interviews

Characteristics			Survey (*n* = 200)	Interview (*n* = 21)
Fathers’ age (years), mean (SD), range			34.2 (4.4), 23.2–49.6	34.6 (4.1), 28.1–40.3
Children’s age (years), mean (SD), range			2.0 (1.1), 0.3–5.1	1.1 (0.6), 0.3–3.1
Father status				
	Expectant		19 (9.5)	3 (14.3)
	1 or more children aged 5 years or younger^1^		161 (80.5)	18 (85.7)
	1 or more children aged 5 years or younger AND			0 (0)
		expecting another child^1^	13 (6.5)	
	A child aged 6 or older AND expecting another			0 (0)
		Child	7 (3.5)	
Age of children				
	Expectant		19 (9.5)	3 (14.3)
	Infant (birth to 1 year)		52 [26]	9 (42.9)
	Toddler (2–3 years)		72 [36]	5 (23.8)
	Preschool (4–5 years)		57 (28.5)	4 (19.0)
Level of Education				
	High school		11 (5.5)	1 (4.8)
	Trade certificate/ TAFE^2^		41 (20.5)	8 (38.1)
	University		148 (74.0)	12 (57.1)
Employment				
	Full-time		169 (84.5)	16 (76.0)
	Part-time / casual		18 (9.0)	3 (14.0)
	Unemployed		3 (1.5)	
	Home Duties		5 (2.5)	1 (4.8)
	Other – (e.g., share trader, travelling)		5 (2.5)	1 (4.8)
BMI				
	Mean (SD), range		27.9 (4.9), 19.4–49.5	26.9 (4.0), 19.6–35.1
	Number (%) categorised as overweight / obesity		142 (71.0)	12 (57.1)
Marital Status				
	Married / De facto		195 (97.5)	21 (100)
	Single / never married		5 (2.5)	0 (0)
Location				
	Metropolitan		123 (61.5)	12 (57.1)
	Regional / rural		77 (38.5)	9 (42.9)
Australian born			176 (88.0)	20 (95.2)
	Aboriginal / Torres Strait Islander		3 (1.5)	0 (0)
Language spoken at home				
	English		187 (93.5)	21 (100)
	Other		13 (6.5)	0 (0)

^1^May also have a child/ren aged 6 and over; ^2^Technical and Further Education

Fathers completing the survey were on average in their mid-thirties, nearly all were married/ defacto, most in full-time employment and nearly three quarters were university educated. The majority were Australian born, spoke English at home, identified as non-Indigenous, were classified overweight or obese and were urban dwelling. The interview sample was mostly representative of the survey sample with the exception that interview participants had on average younger children (1.1 years v 2.0 years) and a lower proportion of interview fathers were university educated (57.0% v 74.0%).

### Survey and interview data

Quantitative and qualitative findings are reported in an integrated format to enhance understanding of the key domains of interest in this study (Table 3).

**Table 3:** Survey domains and Interview themes with illustrative quotes

Domain	Theme	Illustrative Quotes	
1.Role Perceptions	1.1 Active participant	……exercise and level of activity, I think, even if kids aren’t completely	
			mirroring what their parents are doing, they’re still heavily influenced
			by that (F2,postgrad)
		But I like to talk about the different foods. You know what’s the nutritional	
			value. What can you make with it? I enjoy interacting with him about
			that (F7, high school)
	1.2 Supporter	If I can help out and do anything to help her, I will, and it gave me a role to	
			fill I’m giving support (F13, undergrad)^1^
		Paternity leave is something that’s important to me, the women in our	
			workplace have been supported and the blokes are just expected to get
			on with it and keep working through the whole thing (F15 undergrad)^1^
	1.3 Bread winner	In my house growing up, it was the dad goes off to work and the mum stays	
			at home with the kids and all that (F13, undergrad degree)^1^
		I heard unfortunately, quite regularly from dad that a woman’s place was in	
			the kitchen (F5, trade/TAFE)^1^
	1.4 Play partner	….but then, when we got a little bit older, I went to mum for affection and	
			love, and I went to Dad when I wanted some rough housing (F3, Trade/TAFE)
		One reason that I like to promote both nutrition and physical activities, is	
			because my dad also promoted that. He was proactive on, as a kid trying
			different nutritional foods, getting out playing sports (F11, postgrad)
2. Self-efficacy	2.1 Now what	I do remember leaving the hospital and I looked at my partner and we said, is	
			this ours, are we allowed to leave with this child? (F13, undergrad)^2^
		I had difficulty adjusting. I was prepared and feeling confident, but then we	
			had the kid and I had a feeling that I had no idea what I was doing. I was
			just completely overwhelmed (F3, Trade/TAFE) ^2^
	2.2 I’ve got this	You have this added responsibility, and all of a sudden you go, hey, I can do	
			this. Why can I do such and such at work and now, why can’t I look after
			a newborn? And your confidence does go up (F1, trade/TAFE) ^2^
		Physical things is something I should be good at, even if it’s simple, like let’s	
			go and kick a ball. But it might be something that you do as children get
			older, so not necessarily in the formative years (F4, trade/TAFE)
	2.3 Rejection – the great fear	Whenever I cook a new meal for him, I know I have to get out of the house	
			for my wife to feed him. I can’t handle the direct feedback that you get
			from a child who’s not conscious of how you’re feeling (F18, undergrad)
		I like to cook, but you get deflated when they don’t eat it. But when you play	
			you get that feedback that she’s having a good time (F12, trade/TAFE)
	2.4 Confidence	….I had more confidence with the cooking and meal prep. Only because I‘ve	
			had to cook and prepare meals for myself my whole life (F2, postgrad)
	through practice	Our puppy likes it when I rub his belly. So okay, this is slightly more	
			complex [play with baby]. I learn like that. Playing with kids was less
			known. My confidence was lower in that to begin with (F11, undergrad)
3. Support Needs	3.1 Just along for the ride	….they [other dads] don’t have a lot of involvement or buy in, they’re kind of	
			just yeah, just do as I am told kind of thing (F6, trade/TAFE)
		A lot of guys [describing other dads] aren’t going to reach out and say, I don’t	
			understand this. They will just sit in the corner of the maternity ward or
			whatever and just nod or say, I kind of got the hang of that (F2, postgrad)
	3.2 I need a compass	I don’t consider myself an expert in health care. So, I do get confounded by	
			the way, healthcare people communicate things (F8, trade/TAFE)
		There’s empowerment when I got that little bit of info to start off with. I	
			could go to my wife with suggestions, and she would tell me – hey, all
			right thanks, we were going down the wrong path (F10, undergrad)
	3.3 Dads preferences	I like little sort of learning, by little bite size bits of stuff. Videos of nothing	
			more then maybe a minute and thirty seconds (F7, high school)
		The app - baby sensor, it was very helpful. During the pregnancy it was a very	
			useful for breastfeeding and all these other things (F9, undergrad)
	3.4 Bromances	I need like another outlet, being able to have another dad around has	
			absolutely been beneficial (F5, trade/TAFE).
		The podcast Dad’s for dads, it’s kind of like having a beer with your mates,	
			just talking shit. How do you deal with this or that, they call it the Poo
			Army. It’s just like the actual functional things (F13, trade/TAFE)

^1^Role perception quote not directly related to promotion of positive dietary and physical activity behaviours. ^2^Self-efficacy quote not directly related to self -efficacy in promotion of positive dietary and physical activity behaviours

### Perceived paternal role

Survey data and qualitative interviews revealed that fathers were overwhelmingly in strong agreement about the importance of their parental roles (supplementary file 1). From the semi-structured interviews, four themes emerged about how fathers perceive their roles: ‘active participant’, ‘supporter’, ‘bread winner’ and the ‘play partner’ (Table 3). Most fathers fell into one of these roles, however, a small number of fathers also reported taking on multiple roles.

In survey data, more than 90.0% of fathers somewhat/strongly agreed with the importance of role modelling healthy eating and influencing food eaten by babies, toddlers and children. Similarly, more than 90.0% of fathers somewhat/ strongly agreed with the importance of role modelling and participating in physical activity with babies, toddlers and children. Qualitative interviews supported this, with subtheme 1.1 (active participant) describing how fathers were committed to not only role modelling, but also promoting and educating children in positive health behaviours.

Fathers were also committed to being supportive figures with more than 90.0% of survey respondents being in somewhat/strong agreement with the importance of supporting mothers to breastfeed and in establishing and reinforcing rules around screen time and mealtimes. Qualitative interviews highlighted that many fathers had grievances towards current restrictive policies in paternity leave that limited their capacity to be present and supportive at home (subtheme 1.2 - supporter).

A common stress amongst fathers was their commitment to careers while being an available father at home, and for many, this attitude had traditional origins (subtheme 1.3 – bread winner). Many fathers also recounted their own traditional developmental experiences as contributing to their preference for being involved in physical activity rather than nutrition/feeding (subtheme 1.4 – play partner). This was also evident in survey data, with a greater majority of fathers strongly agreeing with the importance of their role in participating in physical activity (91.0 and 92.0% respectively) compared to the provision of nutrition (77.0 and 79.0% respectively) with toddlers (1–2 years) and preschool age children (3–5 years).

### Self - efficacy

Survey data and qualitative interviews revealed that while fathers had overall high self-efficacy, this was susceptible to the various challenges of fatherhood (supplementary file 2). This was reflected in the four themes that emerged from qualitative interviews: ‘now what?’, ‘I’ve got this’, ‘rejection - the great fear’ and ‘confidence through practice’. For some fathers, despite planning and preparation, the arrival of the child resulted in immediate deterioration of self-efficacy (subtheme 2.1 – now what?). In overcoming the initial shock of fatherhood, some fathers spoke of finding reassurance by constructing a plan with their partner, while others experienced improved self-efficacy through participating in mutually enjoyable physical activities with the child (subtheme 2.2 – I’ve got this).

Overall, fathers expressed high self-efficacy with promoting positive nutrition, where for instance, over 90.0% of survey respondents were either somewhat or extremely confident in getting their children to eat enough fruit and vegetables and drink plain water. Fathers also showed high self-efficacy in limiting unhealthy eating behaviours with over 80% of survey respondents being either somewhat or extremely confident in saying no to demands for unhealthy foods such as sweets/ice-cream, chips and cordials. However, greater uncertainty was evident among survey respondents in allowing children to choose how much to eat (23.0% neutral and 72.5% somewhat/extremely confident). Qualitative interviews revealed that when fathers had to learn and apply specific skills in cooking and feeding, their self-efficacy was extremely fragile (subtheme 2.3 – rejection – the great fear). If however, fathers had prior exposure and time to develop these skills, such as via friends/relatives, they reported this opportunity contributed to their high self-efficacy.

Other fathers highlighted improved self-efficacy through learning practical skills in nutrition or playing with their child as the child developed (subtheme 2.4 – confidence through practice). In playing or participating in physical activity, over 90.0% of survey respondents were somewhat or extremely confident about playing with their child, being able to provide a range of play options and get their child to do enough physical activity for health. However, greater uncertainty was evident around TV/ devices where fewer fathers were somewhat/extremely confident in saying no to their child’s demands to watch TV/use a device (80.5%) and to get their child to do active play when they wanted to watch TV/use a device (76.5%).

### Support needs

Four themes emerged about the support needs of fathers: ‘just along for the ride’, ‘I need a compass’, ‘dad’s preferences’ and ‘bromances’. Fathers sought more information about nutrition/feeding in comparison to physical activity and made use of a variety of traditional and contemporary supports. For nutrition/feeding, 72.5% made use of both health professionals and websites, with 6.5% not seeking out any support. While for physical activity, just 19.5% used health professionals and 37.0% websites, with 34.5% not seeking out any support. The support provided by health professionals were well received by fathers with the highest satisfaction ratings (4.31) for nutrition/ feeding and second highest for physical activity (4.44) (Table 4).

**Table 4:** Supports Used

	What types of support have you used to find out information about the nutrition / feeding for your baby / child?		What types of support have you used to find out information about engaging in active play / physical activity you’re your baby child?	
	Number / Percentages of fathers	Average ratings (1 – not beneficial at all, 5 – very beneficial) M(SD)	Number / Percentages of fathers	Average ratings (1 – not beneficial at all, 5 – very beneficial) M(SD)
Family	137 (68.5)	3.65 (1.08)	76 (38.0)	3.77 (1.03)
Female friends	81 (40.5)	3.72 (0.87)	34 (17.0)	4.09 (0.75)
Male friends	63 (31.5)	3.60 (1.04)	35 (17.5)	4.90 (0.75)
Websites	145 (72.5)	3.97 (0.81)	74 (37.0)	3.96 (0.82)
Phone apps	39 (19.5)	3.36 (1.07)	17 (8.5)	3.53 (0.94)
Social media	51 (25.5)	3.39 (0.98)	34 (17.0)	3.82 (0.76)
Parenting ed programs	85 (42.5)	4.06 (0.89)	31 (15.5)	4.29 (0.59)
Health professionals	145 (72.5)	4.31 (0.86)	39 (19.5)	4.44 (0.75)
Childcare staff	47 (23.5)	4.00 (0.84)	24 (12.0)	3.96 (0.91)
Other	19 (9.5)	4.16 (1.20)	13 (6.5)	4.50 (0.91)
No support used.	13 (6.5)		69 (34.5)	

Despite many fathers not actively using supports for physical activity, when fathers were asked what they would like support in, the highest proportion indicated support on participating in active play with children aged 1–2 years (36.0%) and in active play with babies 12 months of age or less (34.0%) (Table 5). This was followed by supports for feeding of babies under 12 months of age and children aged 1–5 years (all 31.5%).

**Table 5:** Supports Wanted^1^

Which of the following areas would you like more information and support in?	Number (Percentages)
Participating in active play with children aged 1–2 years.	72 (36.0)
Participating in active play with babies 12 months of age or less.	68 (34.0)
Feeding babies aged under 12 months (including choice of foods, techniques and times).	63 (31.5)
Feeding of children aged 1–2 years (including choice of foods, techniques and times).	63 (31.5)
Feeding of children aged 3–5 years (including choice of foods, techniques and times).	63 (31.5)
Establishing/ reinforcing rules and routines around TV and screen time, device use with	
Children aged 3–5 years.	63 (31.5)
Participating in active play with children aged 3–5 years.	61 (30.5)
Establishing/ reinforcing rules and routines around TV and screen time, device use with	
Children aged 1–2 years.	52 (26.0)
Supporting my partner to breastfeed.	44 (22.0)
Healthy nutrition information for yourself.	43 (21.5)
I do not need any support	42 (21.0)
Establishing/ reinforcing rules and routines around TV and screen time, device use with	
Babies 12 months of age or less.	41 (20.5)
Healthy physical activity information for yourself.	39 (19.5)
Other	6 (3.0)

^1^ Fathers could select multiple supports

Over half of survey respondents (52.0%) highlighted websites as their preferred option for receiving information, followed by smart phone apps (43.0%) and text messages (42.0%) (Table 6). Traditional forms of face-to-face education were the least preferred option. In interviews, many fathers acknowledged that their involvement in such programs was ‘token ‘as the subject matter was tailored towards the mother only (subtheme 3.1 – just along for the ride). Such experiences contributed to feelings of isolation in fathers, *“everything really sort of revolved around her” (F9, undergrad)*.

**Table 6:** Desired information source^1^

How would you like to receive information about nutrition and active play/ physical activity for your baby/ child?	Number (Percentages)
Websites	104 (52.0)
Smart phone app’s	86 (43.0)
Smart phone technology (i.e. text messages with links to resources)	84 (42.0)
Online training/ education resources	65 (32.5)
Health professionals	63 (31.5)
Social media	50 (25.0)
Face-to-face group session	43 (21.5)
Face-to-face group session with father only classes	31 (15.5)
Face-to-face group session with father only classes and with male facilitators only	10 (5.0)

^1^ Fathers could select multiple information sources

Interviews also revealed some frustration with fathers’ experiences in communicating with health professionals and finding father-specific information on websites. When able to source useable and relevant information however, fathers described positive emotions from obtaining some direction and certainty (subtheme 3.2 – I need a compass). Despite the popularity of websites as an information source, fathers also spoke about the need to have information quickly, without resorting to ‘scatter-gun’ Google searches. Small, ‘bite-sized chunks’ of information and visual demonstrations of relevant skills that can be accessed through Apps or text messages with relevant links were popular amongst fathers (subtheme 3.3 – dads’ preferences).

In survey data, male friends were a popular source of information for nutrition/feeding (31.5%) and active play/physical activity (17.5%). For fathers who did not seek out male friends for support, some stated in interviews that practical and relevant information was a higher priority than the gender of the source. For many fathers however, the company of other dads experiencing the same issues as them was important. For regional / rural based fathers, isolation necessitated the use of father specific podcasts for similar benefits to those fathers who could see other dads face-to-face (subtheme 3.4 - bromances).

## Discussion

This study contributes to the limited pool of knowledge that examines fathers’ perceived roles, self-efficacy and support needs in promoting positive dietary and physical activity behaviours in early childhood. It found that fathers were overwhelmingly aware of the importance of their role and were committed to promoting positive health behaviours. Qualitative interviews provided unique insights into fathers’ experiences and their opinions about what supports and information they need to become engaged with and to promote positive health behaviours to their young children. Fathers have typically found traditional parenting resources to not be suited to their needs [1, 20, 33], therefore this study informs specific strategies that could assist in engaging fathers.

This study was informed by social cognitive theory, which purports that parental behaviours that influence children’s health behaviours occur as a result of interactions between parental role beliefs, self-efficacy and the social and physical environments [24]. There is limited data investigating fathers’ perceptions of their role in dietary behaviours in early childhood, with investigations concentrating on the impact of paternal role modelling, eating behaviours and parenting styles on children’s eating behaviours and obesity risk status [2, 51–55]. The majority of fathers in our study were in acceptance of shared responsibility with mothers in different areas of nutrition, including meal preparation, child feeding and participating in family meals. This aligns with the Australian study of Mallan et al. [14], which found that fathers perceived responsibility for child feeding was positively associated with the frequency of family meals eaten with their children. Fathers in our study were also conscious of the quality of the food they provided, and many took the opportunity to discuss the importance of positive nutrition and limiting exposure to obesogenic foods. More favourable paternal attitudes toward involvement have been associated with greater engagement, warmth and control [56]. Such involvement is conducive to the development of positive dietary behaviours, as shown in the American study of Guerrero et al. [57], which found that children at four-years of age had a lower risk of consuming sugar sweetened drinks if they consumed breakfast more frequently with their father at two-years of age.

To our knowledge, fathers’ perceptions about their role in children’s physical activity in early childhood have not been studied. International studies [16, 58, 59] have reported positive correlations between fathers’ physical activity levels and preschool aged children’s activity levels. In Australia, mixed results were found by Walsh et al. [51], who examined the associations between physical activity levels of fathers and their children with positive associations being observed between light physical activity of healthy-weight fathers and their children at 3.5 years only. Many fathers in our study highlighted a greater affinity and enjoyment in role modelling and participating in physical activity behaviours with their children compared with nutrition. This aligns with stereotypical notions of the father as the primary play partner in early childhood [25], and is in line with development theories that propose fathers are more inclined to engage in exciting and physically stimulating activities with their children than the mother [60]. It is noteworthy however, that as well as enjoying physically engaging with their children, fathers in our study also had strong belief in the benefits of this activity for their children.

The central component of social cognitive theory is self-efficacy [61], which represents one’s beliefs to carry out a course of action successfully and is integral to the interrelationship between environmental, personal and behavioural factors [62]. Research shows that parental self-efficacy is associated with positive child health outcomes [23, 63], and survey data in our study overwhelmingly revealed high paternal self-efficacy in both nutrition and physical activity. However, identified themes in qualitative interviews for self-efficacy (now what?, rejection – the great fear) and support needs (along for the ride, I need a compass), are suggestive of low paternal self-efficacy. Consistent narratives from fathers describing negative influences on their self-efficacy included transitioning to multiple children, fears of rejection (from child), dependence on the partner for direction and needing more father-specific support. Low self-efficacy is likely indicative of first-time fathers in our sample, newly exposed to the pressures of caring for a child and also needing to adapt to new pressures as the child grows. Fragile self-efficacy has been noted in a rare study exploring paternal self-efficacy for promoting obesity-protective diets in early childhood by Walsh et al. [64]. This study highlighted the need for support as a means of firstly increasing, and secondly to prevent decreasing, paternal self-efficacy through the stages of infancy and early childhood.

The majority of fathers in our study were open to accessing social support and information/resources when needed. In many cases, this was in conflict with more traditional paternalistic values they were exposed to in their own development. Fathers described undergoing a learning process in transitioning to fatherhood that made them more amenable to using social support. Their willingness to seek and offer support is consistent with other research reporting a shift in men’s attitudes post fatherhood, to identifying themselves as a father first and foremost, with a subsequent emergence of appropriate help-seeking behaviours [65]. Despite wanting supports however, many fathers experience frustration when unable to locate what they need, thereby increasing the chances of disengagement [20]. Some fathers also reported that they felt like outsiders when dealing with trusted professionals, such as hospital staff or doctors. Such experiences have also been reported in qualitative findings of fathers dealing with medical staff [34], and are representative of men feeling ignored and dependent on the mother’s guidance [66], thereby increasing the chances of deteriorating self-efficacy.

Fathers in our study also experienced significant challenges when navigating online resources to find useful information. This is in line with previous Australian based qualitative research [33], where fathers of preschool aged children found searching for information online to be a frustrating endeavour. Consistent with other forms of information, fathers also found that the information was heavily mother centric and offered little to their specific needs. Of online technology that fathers found informative and supportive, fathers highlighted the improved confidence that came with resources that resonated with them. This is consistent with Australian research into the effects of supportive text messages on new fathers mental health, which showed these messages helped in lowering isolation, managing the pressures of fatherhood and assisting with their relationship with their partners [20].

Strengths of this study included the mixed methods design, which allowed fathers’ perspectives to be explored in more detail through qualitative interviews, providing a deeper understanding of fathers’ experiences and viewpoints. This study included fathers experiencing differing stages of early childhood, from expectant to experienced and therefore provides unique insights to a limited evidence base of the positive contributions that fathers can have throughout early childhood. The findings add support to similar research that has found traditional forms of parental support are failing to equip fathers with the information and strategies they need in a stressful period of fatherhood [20, 33]. This study was limited by a sample of mostly higher educated, predominantly Australian-born fathers who by their interest in this study, were likely to be committed and motivated paternal figures. This may have contributed to social desirability bias in both quantitative and qualitative responses. Notwithstanding the difficulties in recruiting fathers of lower socioeconomic status in parenting research, future studies would benefit from input from father’s representative of a more varied sample. Future research will benefit from further delineation of potential differences in fathers’ beliefs, self-efficacy and support needs according to differing developmental stages from expectant fathers to preschool age children as well as the impact of differing number of children and potential impacts of blended families.

## Conclusion

This study has shown that fathers were overwhelmingly aware of the importance of their role, were committed and had high self-efficacy in being a positive influence on their children’s health through promoting optimal nutrition and physical activity behaviours. However, many fathers’ beliefs and self-efficacy were susceptible to change and deterioration. Fathers highlighted their experiences of a lack of access to resources and information that they could understand, resulting in perceptions of isolation and becoming impediments to promoting positive health behaviours to their children. Providing fathers with accessible, father-specific, reputable information should be considered as a strategy to support fathers to promote positive health behaviours to their children with life-long benefits.

### Electronic supplementary material

Below is the link to the electronic supplementary material.

**Supplementary Material 1: Supplementary Table 1** Fathers Perceived Roles; **Supplementary Table 2**. Fathers’ Self-efficacy; **Supplementary File 3**. Interview Guide

## Data Availability

The data sets used and/or analysed during the current study are available from the corresponding author on reasonable request.

## Abbreviations

SCT: Social Cognitive Theory
INFANT: Infant Feeding Activity and Nutrition Trial
PASSS: Physical Activity and Social Support Scale
BMI: Body Mass Index
IBM: International Business Machines
TAFE: Technical and Further Education
PLS: Plain Language Statement
