# Supplementary material for: Fathers’ perceived role, self-efficacy and support needs in promoting positive nutrition and physical activity in the first 2000 days of life: a mixed methods study
Source: Int J Behav Nutr Phys Act. 2024 Feb 26;21:23. doi: 10.1186/s12966-024-01575-w (PMC10895814; doi:10.1186/s12966-024-01575-w)
Supplement: Supplementary file 1 — Supplementary Material 1: Supplementary Table 1 Fathers Perceived Roles; Supplementary Table 2. Fathers’ Self-efficacy; Supplementary File 3. Interview Guide [file 12966_2024_1575_MOESM1_ESM.docx]

**Supplementary Files**

**Supplementary File 1**: Fathers Perceived Role Frequency Table

|  | | Strongly / somewhat  Disagree  Number  (Percentages) | Neutral | Somewhat  Agree | Strongly  Agree |
| --- | --- | --- | --- | --- | --- |
| The way in which a dad engages with his | | 7 (3.5) | 9 (4.5) | 44 (22.0) | 140 (70.0) |
|  | baby in the first 6 months has |  |  |  |  |
|  | Important life-long effects on the child |  |  |  |  |
| It is essential that the dad role models | | 2 (1.0) | 8 (4.0) | 53 (26.5) | 137 (68.5) |
|  | healthy eating to the baby/ child |  |  |  |  |
| Dads should be as involved as the | | 2 (1.0) | 2 (1.0) | 28 (14.0) | 168 (84.0) |
|  | mothers in establishing and reinforcing |  |  |  |  |
|  | mealtimes rules |  |  |  |  |
| Dads play a central role in supporting | | 4 (2.0) | 8 (4.0) | 67 (33.5) | 121 (60.5) |
|  | mothers to breastfeed |  |  |  |  |
| Dads have an important role to play in | | 0 | 12 (6.0) | 63 (31.5) | 125 (62.5) |
|  | influencing what food is provided to |  |  |  |  |
|  | their baby at 12 months and less |  |  |  |  |
| Dads have an important role to play in | | 0 | 6 (3.0) | 40 (20.0) | 154 (77.0) |
|  | influencing what food is provided to |  |  |  |  |
|  | their toddler aged 1-2 years |  |  |  |  |
| Dads have an important role to play in | | 0 | 7 (3.5) | 35 (17.5) | 158 (79.0) |
|  | influencing what food is provided to |  |  |  |  |
|  | their pre-schooler aged 3-5 years. |  |  |  |  |
| It is essential that dad role-models being | | 2 (1.0) | 5 (2.5) | 34 (17.0) | 159 (79.5) |
|  | physically active to the baby/ child |  |  |  |  |
| Dads should be as involved as the | | 1 (0.5) | 2 (1.0) | 14 (7.0) | 183 (91.5) |
|  | mothers in establishing and reinforcing |  |  |  |  |
|  | screentime rules |  |  |  |  |
| Dads have an important role to play in | | 1 (0.5) | 1 (0.5) | 28 (14.0) | 170 (85.0) |
|  | participating in active play with their |  |  |  |  |
|  | baby aged 12 months of age or less. |  |  |  |  |
| Dads have an important role to play in | | 0 | 0 | 18 (9.0) | 182 (91.0) |
|  | participating in active play with their |  |  |  |  |
|  | toddler at 1-2 years. |  |  |  |  |
| Dads have an important role to play in | | 0 | 1 (0.5) | 15 (7.5) | 184 (92.0) |
|  | participating in active play with their |  |  |  |  |
|  | pre- schooler aged 3-5 years. |  |  |  |  |

**Supplementary File 2**: Fathers Self-efficacy Frequency Table

|  | | | Not / slightly confident  Number  (Percentages) | Neutral | Somewhat confident | Extremely confident |
| --- | --- | --- | --- | --- | --- | --- |
| Get my child to eat enough | | | 18 (9.0) | 17 (8.5) | 105 (52.5) | 60 (30.0) |
|  | vegetables (this does not include | |  |  |  |  |
|  | potatoes or potato chips) | |  |  |  |  |
| Get my child to drink plain water (with | | | 3 (1.5) | 7 (3.5) | 51 (25.5) | 139 (69.5) |
|  | no flavours or juice added) | |  |  |  |  |
| Say ‘no’ to my child’s demands for | | | 6 (3.0) | 13 (6.5) | 52 (26.0) | 129 (64.5) |
|  | soft-drinks, fruit juice, cordials and | |  |  |  |  |
|  | other sweetened drinks | |  |  |  |  |
| Say ‘no’ to my child’s demands for | | | 16 (8.0) | 17 (8.5) | 75 (37.5) | 92 (46.0) |
|  | potato chips and similar foods | |  |  |  |  |
| Say no to my child’s demands for | | | 14 (7.0) | 18 (9.0) | 82 (41.0) | 86 (43.0) |
|  | sweet snacks, confectionary, | |  |  |  |  |
|  | lollies and/ or ice-cream | |  |  |  |  |
| Get my child to eat a wide range of | | | 15 (7.5) | 11 (5.5) | 91 (45.5) | 83 (41.5) |
|  | foods. | |  |  |  |  |
| Get my child to eat enough fruit (this | | | 7 (3.5) | 9 (4.5) | 62 (31.0) | 122 (61.0) |
|  | does not include fruit juice) | |  |  |  |  |
| Allow my child to choose how much | | | 9 (4.5) | 46 (23.0) | 69 (34.5) | 76 (38.0) |
|  | s/he eats | |  |  |  |  |
| Get my child to eat a variety of | | | 16 (8.0) | 11 (5.5) | 98 (49.0) | 75 (37.5) |
|  | fruit/vegetables | |  |  |  |  |
| Eat meals with my child on most | | | 16 (8.0) | 21 (10.5) | 71(35.5) | 92 (46.0) |
|  | days | |  |  |  |  |
| Talk with people who care for my | | | 8 (4.0) | 23 (11.5) | 68 (34.0) | 101 (50.5) |
|  | child about what to feed him /her | |  |  |  |  |
| Talk with people who care for my | | | 5 (2.5) | 15 (7.5) | 67 (33.5) | 113 (56.5) |
|  | child about encouraging active | |  |  |  |  |
|  | play (ie walking, playing outside) | |  |  |  |  |
| Talk with other people who care for | | | 10 (5.0) | 23 (11.5) | 69 (34.5) | 98 (49.0) |
|  | my child about limiting the amount | |  |  |  |  |
|  | of TV s/he watches | |  |  |  |  |
| Turn off the TV during mealtimes | | | 17 (8.5) | 20 (10.0) | 50 (25.0) | 113 (56.5) |
| Say 'no' to my child’s demands to | | | 17 (8.5) | 22 (11.0) | 61 (30.5) | 100 (50.0) |
|  | watch TV/ use devices | |  |  |  |  |
| Get my child to do some active play | | | 12 (6.0) | 35 (17.5) | 85 (42.5) | 68 (34.0) |
|  | when s/he wants to watch TV/ | |  |  |  |  |
|  | use a device | |  |  |  |  |
| Provide my child with a range of | | | 4 (2.0) | 10 (5.0) | 83 (41.5) | 103 (56.5) |
|  | active play/ physical activity | |  |  |  |  |
|  |  | options |  |  |  |  |
| Get my child to do enough active | | | 7 (3.5) | 10 (5.0) | 78 (39.0) | 105 (52.5) |
|  | play/ physical activity for health | |  |  |  |  |
| Keep my child entertained without | | | 9 (4.5) | 22 (11.0) | 90 (45.0) | 79 (39.5) |
|  | using TV/ devices | |  |  |  |  |
| Play with my child (not including | | | 3 (1.5) | 7 (3.5) | 52 (26.0) | 138 (69.0) |
|  | computer games?) | |  |  |  |  |

**Supplementary File 3:** Interview Guide

- Thank you for completing the questionnaire. This follow-up interview will give us the opportunity to expand on some of the topics in the questionnaire and will go for approximately 30-60 minutes.
- I just wanted to quickly go through the consent form and confirm you’re willing to be interviewed. Do you mind if I record your consent? Also, please remember that you don’t have to answer the questions if you don’t want to for any reason and let me know if you want to take a break.
- There will be no right or wrong answers, so please feel free to answer as you see fit.
- Any last questions before we get started?
- Perceived paternal role:
- 'To start with could you tell us a bit about your experiences of being a dad?'
- What role do you think Dads play in influencing their children's development under the age of 5? Prompt: Traditional stereotypical attitudes are that fathers are providers and mothers are nurturers. Agree / disagree?
- What role do you think fathers play in helping their child learn healthy eating habits? Follow-on: And how about the mothers?
- What has been your experiences in feeding your children? Prompts: How involved have you been? How does your role compare to that of your wife/partner?
- What role do fathers play in helping their kids learn to be active? Follow-on: And how about mothers? Prompts: How involved have you been in playing with your kids? How does that compare to that of your wife/partner? Explore – fathers as play partner?
- What activities do you prefer participating in with your child? Ie Feeding / active play?
- Self-efficacy:
- Particularly for new fathers, the early childhood phase can be very stressful. How confident are you / will you be in spending time with your children aged 5 and under? Prompt: Explore participants specific situation – Did you/ will you have trepidation? Experiences with other children in that age? Prefer certain phase. Ie baby, toddler or later age, has your confidence improved over time?
- How confident do you feel in engaging (or do you think you will feel – expectant fathers) in physical activity with your child, can you tell me why that is the case? Follow-on: Is this confidence the same as feeding your child and being involved in his food choices? (If confidence levels are different) - Can you tell me more about what is affecting your confidence in this area but not in active play?
- What are some of the barriers or things that might make it more difficult for you to be involved with feeding your child (such as planning meals, active feeding, cooking and or eating with the family)? What would help or make this easier?
- In terms of active play/ physical activity, what are some of the barriers or things that might make it more difficult for you to be involved with playing or taking part in a physical activity with your child? What would help or make this easier?
- I think every time I go to my local supermarket, I see young children demanding that their parents buy them lollies or unhealthy foods like ice-cream. How confident are you (or will be) in saying no to your child’s demands in situations like this for foods that you know are unhealthy? Follow on: Explore participants specific situation - Experiences with other children in that age? Experiences where children may want a quick snack at home and your busy doing something else and just want to keep the occupied for a short time. How do you children respond to the foods you choose to eat?
- We are living in a very different world to the one where you and I grew up in, with addictive devices and the internet at a fingertip away from children. How confident are you that you will be able to say no to your child’s demands to use a devices / watch TV and do some active play/ physical activity instead. Follow-up: In a similar situation to the scenario in the previous question where you may be under time stress and need to organise some dinner or attend to some work. Do you still have confidence that you will be able to give something other than a device /TV to occupy their time when they are demanding it?
- How do you feel about your own physical activity levels? Follow-on: In what ways, if at all, do you think this influences how active your kids are? What barriers have you experienced in role modelling physically activity to your child.
- Support Needs:
- What have been your experiences with getting information or advice in the areas of nutrition and active play / physical activity? Follow-on: Have you looked for specific resources and were you able to find the information you wanted and did they meet your needs? If you couldn’t find it, or did you give up and / or tried to work things out yourself? Where/ how did you look?
- What were your reasons for turning to this type of resource? Prompt: ie If previously highlighted social media / facebook etc. further explore was convenience / anonymity important to you? If family member – was trust important?
- Considering the resources / forms of support that you used, have you had the opportunity to mix with and hear the opinions / experiences of fathers? How did you find that? Follow-on: If you haven’t as yet had the chance to mix with other fathers in a similar position to you, how do you think you would find that?
- Have you attended any parenting groups/sessions? What were your experiences / perceptions? Follow-up: How many fathers were involved? Did you have the chance to mix with father only classes? Your experiences of these?
- Follow-on: If you haven’t attended any parental training / education programs, what would draw you to attend? Prompt father only classes and / or male only teachers be a positive factor in your decision to attend?
- What kind of information or support would you like to get as a Dad to help you with feeding your children and promoting good nutrition?
- What type of information and support would you find useful in encouraging your kids to be physically active
- Is there anything else that you would like to add?
